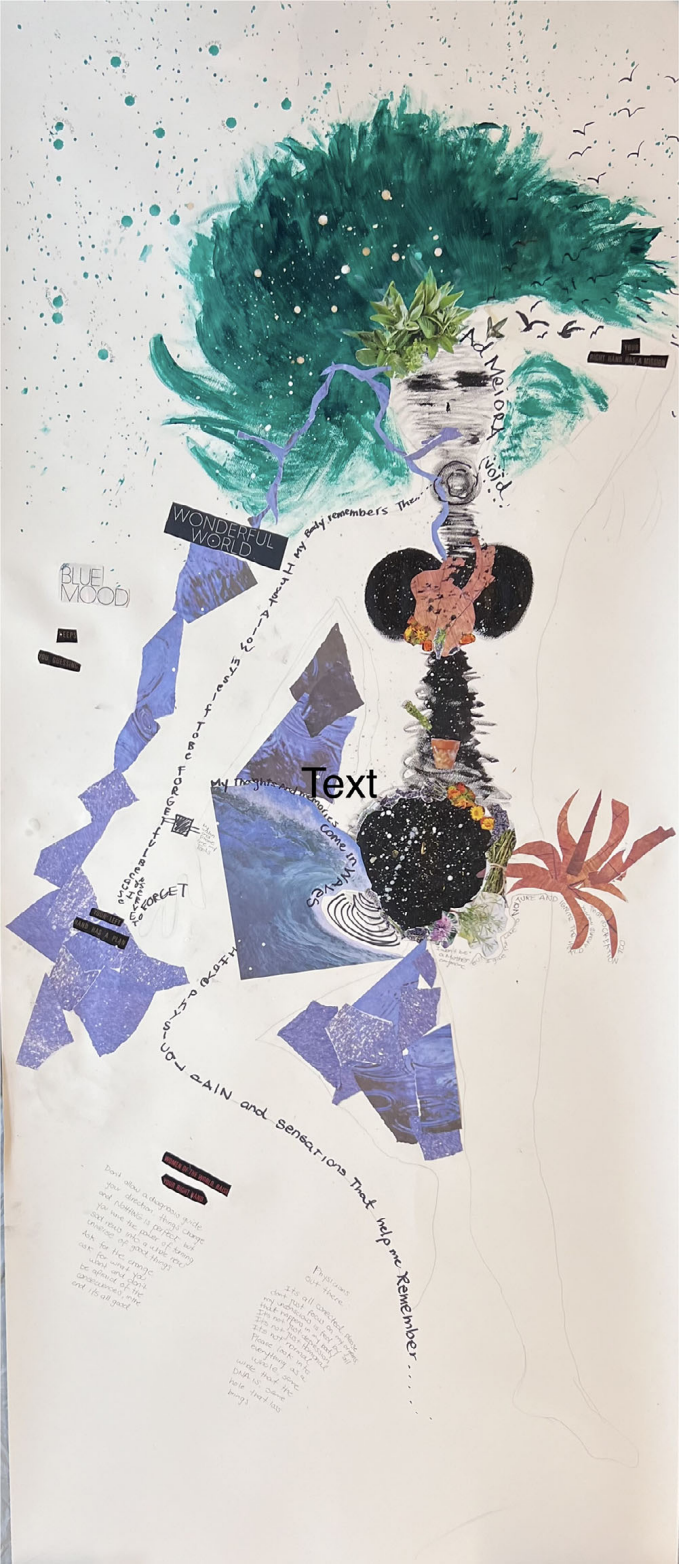# The many menopauses: Subjective Cognitive Decline in younger women with bilateral oophorectomy

**DOI:** 10.1002/alz70857_101078

**Published:** 2025-12-24

**Authors:** Gillian Einstein, Jana Galley, Lucy Muir, Laurice Karkaby, Denise Gastaldo, Andrea Charise, Hemangi Shroff

**Affiliations:** ^1^ University of Toronto, Toronto, ON, Canada; ^2^ Toronto Arts Foundation's Neighbourhood Arts Network initiative, Toronto, ON, Canada

## Abstract

**Background:**

There are many menopauses varying in their trajectories toward aging and some, dementia. Surgical menopause from early‐life bilateral ovarian removal (BSO), marked by immediate cessation of ovarian hormone production, notably 17‐b‐estradiol (E2), has perhaps the most severe trajectory. Large cohort data show increased late‐life Alzheimer's disease risk and steeper cognitive decline compared to spontaneous menopause (SM); smaller studies within five years post‐BSO show decreased verbal episodic and spatial working memory vs. age‐matched controls.

**Objectives:**

To determine how younger, middle‐aged women (average age 43 at BSO; 48 at study) perceive their memory after BSO.

**Method:**

Using qualitative interviews and body map/story‐telling (BMST), we collected women with BSO's stories about their memory: if they perceived a decline, how they felt, about it, and the corporeal experiences of memory problems.

**Results:**

In interviews with younger women with BSO, many told us they experience memory problems. Many of these participants also had either verbal or spatial memory decrements within five years post‐BSO. They reveal those memory losses to be in word‐finding, remembering people's names, and where they place things. Preliminary themes emerging from BMST were: (i) Chaos around the head; (ii) Memory Tools and (iii) Menopause affects daily life: Ovarian removal in a menopause milieu.

**Conclusion:**

SCD may be an immediate outcome of BSO prior to age 50. It prompts women to feel old, express concerns about their memory, and use memory tools. SCD+ generally includes women in SM and includes several risk factors; we might consider including BSO prior to age 50.